# Female breast cancer subtypes in the Romagna Unit of the Emilia-Romagna cancer registry, and estimated incident cases by subtypes and age in Italy in 2020

**DOI:** 10.1007/s00432-023-04593-6

**Published:** 2023-03-15

**Authors:** Emanuele Crocetti, Alessandra Ravaioli, Orietta Giuliani, Lauro Bucchi, Rosa Vattiato, Silvia Mancini, Federica Zamagni, Benedetta Vitali, Chiara Balducci, Flavia Baldacchini, Fabio Falcini

**Affiliations:** 1 Romagna Unit of the Emilia-Romagna Cancer Registry, Romagna Cancer Institute, IRCCS Istituto Romagnolo per lo Studio dei Tumori (IRST) Dino Amadori, Forlì, Meldola, Italy; 2 Local Health Authority, Cancer Prevention Unit, Forlì, Italy

**Keywords:** Breast cancer, Cancer registry, Hormone receptor (HR), Human epidermal growth factor receptor 2 (HER2), Population-based, Subtypes

## Abstract

**Purpose:**

The aim of this study was to estimate the Italian burden of incident breast cancer (BC) by subtypes, according to the distribution of hormonal receptor (HR) status and expression of human epidermal growth factor 2 (HER2).

**Methods:**

Female breast cancers incidence in the Romagna Unit of the Emilia-Romagna registry (*n*. 10,711) were grouped into: HR+ /HER2–, HR+ /HER2+ , HR–/HER2+ , HR–/HER2– and missing, and by age: < 50, 50–69 and 70+ years. Data were compared with other published Italian population-bases series before using them for national estimates. We used national and regional numbers of expected breast cancers published by the Italian network of cancer registries considering the age- and geographic-specific variation of the Italian population.

**Results:**

Overall, 70.7% of incident BC cases are expected to be HR+ /HER2-, 8.5% HR+ /HER2+ , 7.5% HR-/HER2-, 4.1% HR-/HER2+ and 9.3% missing. The global ranking is similar across age-groups but with age-specific differences. The proportion of missing was around 3-times lower than in the other Italian published population-based series and similar to the SEER one. In Italy, are estimated 38,841 HR+ /HER2- breast cancer cases, 4665 HR+ /HER2+ , 4098 HR-/HER2-, 2281 HR-/HER2+ , and 5092 not specified. Numbers by age-group were provided.

**Conclusions:**

The present estimates relied on high-quality population-based data and provide a clinically relevant information on the burden of breast cancer subtypes. These data will support the planning of therapy needs for oncologists, decision-makers, and all other stakeholders.

## Introduction

Worldwide female breast cancer (BC) is, by far, the main cause of oncological deaths and the most frequently diagnosed cancer among women (Ferlay et al. 2020); Italy does not make an exception to such pattern (AIOM 2021a). Over the years, BCs have been split into different subtypes according to the combination of hormonal receptor (HR) status, including estrogen and progesterone receptors, and the expression of human epidermal growth factor 2 (HER2), as well as other bio-markers. (Perou et al. 2000; Goldhirsch et al. 2011).

The main molecular subtypes defined by immune-histochemistry, based on HR and HER2 status only (other classifications include other markers) (Goldhirsch et al. 2013; Guiu et al. 2012), are four: HR+ /HER2– (i.e., approximating Luminal A; hereafter Luminal A-like), HR + /HER2 + (i.e., approximating Luminal B; hereafter Luminal B-like), HR–/HER2+ (HER2 enriched), and HR–/HER2– (triple-negative). (Howlander et al. 2014; Johansson et al. 2019).

The distinct BC subtype determines differences in the medical therapy approach (hormonal therapy, target therapy, chemotherapy, or immunotherapy) and a different prognosis. (AIOM 2021b; Howlander et al. 2018).

Periodically, the Italian network of population-based cancer registries (Airtum) provides updates estimating the burden of cancers in the country (AIOM-Airtum 2020), but a comprehensive picture of BC subtypes is not available yet. Moreover, the available data on BC subtypes from Italian population-based cancer registries refer to past years and/or have a huge proportion of cases with missing information (Caldarella et al. 2011; Minicozzi et al. 2017; Tagliabue et al. 2021).

The aim of this paper is to fill this gap evaluating the BC subtypes distribution in a high-quality population-based cancer registry (CR) and projecting the estimation to Italy, for groups of ages.

## Methods

We analyzed the age distribution (< 50, 50–69 and 70 and more years) and subtypes (HR+ /HER2-, HR+ /HER2+ , HR-/HER2 + , HR-/HER2-, unknown) of BC cases in the Romagna Unit of the Emilia-Romagna Cancer Registry, during 2008–2017.

The population-based cancer registry is located in Northeast Italy covering a population of 1,122,149 inhabitants (on January 1, 2018). Its data passed the quality evaluation of the last six editions of Cancer Incidence in 5 Continents of the International Agency for Research on Cancer (Bray et al. 2017), they are accredited by Airtum (www.registri-tumori.it) and also included in the European Cancer Information System of the European Commission (https://ecis.jrc.ec.europa.eu).

The registry’s data have been extensively analyzed for BC studies (Bucchi et al. 2019, 2021; Giuliani et al. 2016; Musolino et al. 2016; Ravaioli et al. 2018).

The age and subtypes specific distribution observed in the Romagna Unit of the Emilia-Romagna Cancer Registry, was applied to the region-specific age-classes distribution and summed up for producing the subtype and age estimated distribution of Italian BC cases.

In particular, we used the estimated overall number of female breast cancers newly diagnosed in Italy in 2020 published by Airtum (AIOM-Airtum 2020). The gran total for 2020 was regionally distributed with the same proportions estimated for 2019 (AIOM-Airtum 2019).

The age (< 50, 50–69, 70+ years) distribution of BC cases in the Italian population-based series of over 100,000 cases included in Cancer Incidence in 5 Continents XI was: in the registries of the north (< 50 = 21.6%, 50–69 = 42.5% and 70+ years = 35.9%), center (23.0%, 44.7%, and 32.4%), and south (26.2%, 42.9%, 30.9%) (Bray et al. 2017). Therefore, the age-specific values for the three geographical areas were applied to the pertinent regions, namely for north (Emilia-Romagna, Friuli Venezia Giulia, Liguria, Lombardy, Piedmont, Trentino Alto Adige, Valle d’Aosta and Veneto), center (Lazio, Marche, Toscana and Umbria) and south (Abruzzo, Basilicata, Calabria, Campania, Molise, Puglia, Sardinia and Sicily). Each estimated combination of age and BC subtypes expresses the proportion of the overall BC estimated number (54,976) for the specific weight of age (geographically weighted), and subtype.

Estimated numbers are presented for Italy as frequencies and percentages.

## Results

Table 1 shows the age-specific and overall distribution of 10,711 cases of BC incident in 2008–2017 in the population resident in the area of the study.

**Table 1:** Romagna Unit of the Emilia-Romagna cancer registry: distribution of female breast cancer cases incident during 2008–2017 by subtype and age. Comparison with other published Italian population-based case series and SEER one

	Age (years)	Subtype					
		HER2-/HR+ *N*. (%)	HER2+ /HR+ *N*. (%)	HER2+ /HR- *N*. (%)	HER2-/HR- *N*. (%)	Unknown *N*. (%)	Total
	< 50	1587 (68.2)	290 (12.5)	121 (5.2)	216 (9.3)	115 (4.9)	2329
Present study	50–69	3343 (73.9)	391 (8.6)	225 (5.0)	346 (7.6)	222 (4.9)	4527
	70 +	2,633 (68.3)	215 (5.6)	92 (2.4)	230 (6.0)	685 (17.8)	3855
	Tot	7563 (70.6)	896 (8.4)	438 (4.1)	792 (7.4)	1022 (9.5)	10,711
Caldarella et al. (2011)		1045 (52.7)	232 (11.7)	90 (4.5)	120 (6.0)	497 (25.1)	1984
Minicozzi et al.^b^ (2017)		2309 (51.3)	464 (10.3)	224 (5.0)	384 (8.5)	1116 (24.8)	4497
Tagliabue et al. (2021)		3798 (43.0)	1093 (12.4)	354 (4.0)	490 (5.5)	3095 (35.1)	8831
SEER^a^		(68)	(10)	(4)	(10)	(7)	337,102

^a^2015–2019, https://seer.cancer.gov/statfacts/html/breast-subtypes.html

^b^In the original analysis the subtypes were classified using the system which includes, out of HR and HER2 status, also the Ki67 index. Cases have been reclassified according to the present criteria

The most frequent subtype, in all the three age-groups, was Luminal A-like (70.6%), followed by Luminal B-like (8.4%) which showed a decreasing trend with increasing age (from 12.5% among women aged < 50 years to 5.6% in those of 70 years or older). The third most frequent subtype was the triple negative, 7.4%, and the least frequent the HER2 + enriched (4.1%), in particular among the oldest women (2.4%).

Moreover, for 9.5% of the BC cases the information on HR and/or HER2 was not available. In particular, out of the 1022 missing cases, 575 (56.3%) had the information on HR status (512 HR + and 63 HR-) and 27 (2.6%) on HER2 (21 negative and 6 positive), while for 299 (41.1%) each information was missing.

The proportion of subtype unknown changed across ages being 4.9% below and 17.8% above 70 years. The overall mean age at diagnosis of BC cases with missing information for subtype was 74 years, while for not missing 62, and by age-groups 44 vs 44 for BC in women < 50 years, 60 vs 60 for 50–69 years, and 84 vs 78 in women 70 years or older.

In addition, the proportion of metastatic cases at diagnosis for BC patients without subtype information was 16.6% vs 5.0% for the others.

In Table 1 the subtypes distribution is also presented for the other Italian population-based series published since now (Caldarella et al. 2011; Minicozzi et al. 2017; Tagliabue et al. 2021) and for the US SEER (https://seer.cancer.gov/statfacts/html/breast-subtypes.html). The previous Italian series confirmed the ranking among the subtypes observed in the current study but they had a very high proportion of missing information (ranging from 25 to 35%). The US SEER distribution seemed quite close to the present series, including for the missing class.

Table 2 shows the estimated number of incident BC in Italy in 2020 according to the immune-histochemical subtype and age-group.

**Table 2:** Estimated distribution of incident female breast cancers by age-groups and subtypes in Italy in 2020

Age	Breast cancer molecular subtypes					
Years	HER2-/HR +	HER2 + /HR +	HER2 + /HR-	HER2-/HR-	Unknown	Total
	*n.*/%	*n*./%	*n.*/%	*n.*/%	*n*./%	*n.*
< 50	8666	1584	661	1180	628	12,718
	68.1%	12.5%	5.2%	9.3%	4.9%	
50–69	17,479	2044	1176	1809	1161	23,669
	73.8%	8.6%	5.0%	7.6%	4.9%	
70+	12,696	1037	444	1109	3303	18,589
	68.3%	5.6%	2.4%	6.0%	17.8%	
All	38,841	4665	2281	4098	5092	54,976
	70.7%	8.5%	4.1%	7.5%	9.3%	

In 2020 in Italy, out of the estimated 54,976 newly diagnosed BC, 38,841 (70.7%) are expected to be Luminal A-like, 4665 (8.5%) Luminal B-like, 4098 (7.5%) Triple-negative, 2281 (4.1%) HER2 enriched, and 5092 (9.3%) not specified.

Among younger women (< 50 years) 8666/12,718 (68.1%) BC cases are estimated to be Luminal A-like, 1584 (12.5%) Luminal B-like, 1180 (9.3%) Triple-negative and 661 (5.2%) HER2 enriched. In the age-group 50–69 years, the estimated burden of Luminal A-like is 73.8% of the overall 23,669 expected cases. Moreover, we expect 2044 (8.6%) Luminal B-like, as well as 1809 (7.6%) Triple-negative and 1176 (5.0%) HER2 enriched. Lastly, among women aged 70 years and over, 18,589 new BC case are predicted for 2020 in Italy, of which 12,696 (68.3%) Luminal A-like, 1037 (5.6%) Luminal B-like, 1109 (6.0%) Triple-negative, and 444 (2.4%) HER2 enriched. Moreover, for 5092 BC cases (9.3%), respectively 4.9%, 4.9% and 17.8% in the three age-groups, the information is missing.

## Discussion

Breast cancer subtype is an essential variable for managing the medical therapy. (AIOM 2021b) As far as we know, up to now no national estimates of BC subtypes are available for Italy.

The strength of this analysis is to be grounded on reliable data from Italian population-based cancer registries, that have no selections of patients due to old ages, advanced stage at diagnosis, or eligibility to treatment, as may happen in clinical series.

Italian cancer registries belong to Airtum which evaluates the quality of member registries data (Airtum 2013). In the present study the overall number of BC cases incident in 2020 was provided by Airtum (AIOM-Airtum 2020). This gran total was ascribed to each region according to the relative ratio used by Airtum in the previous year (AIOM-Airtum 2019). This choice seems reasonable considering that no major changes neither in incidence nor in the size of the population are expected in a short time frame (AIOM-Airtum 2018).

BC incidence varies in Italy decreasing from north to south. (Andreano et al. 2019) Moreover, the age-structure of BC cases changes as well with a younger population in the south. Therefore, it was necessary to consider the age-structure of BC cases across the Country. For this reason, we relied on a huge Italian population-based series of 35 cancer registries accepted for publication by the International Agency for Research on Cancer in Cancer Incidence in 5 Continents XI (Ferlay et al. 2020), splitting them according to the geographical location (north, center, south). The gradient of incidence of BC is quite strong between north-center (almost similar) and south, but negligible within the macro areas (Andreano et al. 2019).

Finally, we estimated the Italian expected figures summing up region- and age-specific numbers of BC using the age-specific proportions of subtypes observed in the ten-year BC case-series of the Romagna Unit of the Emilia-Romagna Cancer Registry. Immuno-histochemical markers are not included among the compulsory variables to be collected by cancer registries, yet, neither by Airtum, nor by the European Network of Cancer Registries (ENCR). However, the Romagna Unit of the Emilia-Romagna Cancer Registry is managed by researchers particularly interested in BC epidemiology (Bucchi et al. 2019; Bucchi e al. 2021; Giuliani et al. 2016; Musolino et al. 2016; Ravaioli et al. 2018). Therefore, information about BC, including subtypes, are collected more extensively than in other CRs, in fact, the proportion of missing subtype is around from 2 to threefold lower than in other Italian published studies (Minicozzi et al. 2017; Caldarella et al. 2011; Tagliabue et al. 2021).

Notwithstanding, 9.5% of the analyzed cases are not characterized for both HR and HER2, a proportion not far from the 7% measured in the US SEER (https://seer.cancer.gov/statfacts/html/breast-subtypes.html). It is plausible to assume that at least part of these cases has been tested for these markers but the Registry’s staff did not succeed in collecting the information, but for 58.9% of them at least one out of two markers was available. In any case, this may be the case of patients who had the analysis in another region. However, the Emilia Romagna region has one of the lowest health migration rates for cancer in the Country. In addition, BC patients who spent their full diagnostic and therapeutic path in private services, outside the universal public health system, are not under cancer registry control.

At the same time, some of the BC cases may not perform such tests for the very advanced stage at diagnosis, or old age. In fact, we documented a higher proportion of metastasis at diagnosis in patients with missing markers’ information than in the others and an older mean age at diagnosis. Moreover, the percentage of missing data among oldest women are threefold than for the youngest and middle-aged ones. Finally, the presence of comorbidities, or general conditions may not allow systemic therapy and therefore in-depth diagnostic analysis.

The choice to use the Romagna Unit of the Emilia-Romagna Cancer Registry data, which includes the subtype unknown, seemed tenable for estimating a real-world scenario. Otherwise, the option of using one of the available Italian data (Minicozzi et al. 2017; Caldarella et al. 2011; Tagliabue et al. 2021) limited to not missing information would have introduced a bias considering, for example, the relationship between missing data and age.

The basic assumption of the present study was that the age-specific proportions of BC subtypes were stable across the Country. This may not be the case. On the one hand, geographic differences in the prevalence of epidemiologic risk factors across Italy are diminishing (Giorgi Rossi et al. 2020). On the other hand, however, heterogeneity in the prevalence of mammography screening might cause another type of bias in the extrapolation of data from a region of the north—where uptake of mammography screening programmes is higher—to the south of the country, because screen-detected BCs include a larger subset of Luminal A subtypes than is observed among symptomatic BCs. However, the difference is modest in relative terms, for example: 81.9% versus 70.74% according to the study by Farshid and Walters (Farshid and Walters 2018). Equally important, the north–south decreasing gradient in overall prevalence of mammography use, pooling organized and opportunistic screening practice, has become less steep (Giorgi Rossi et al. 2020) and is currently not pronounced, except for the regions of Campania and Calabria (ONS 2022).

In fact, the other Italian published papers which analyzed BC subtypes presented similar proportions among the Tuscany cancer registry (central Italy) (Caldarella et al. 2011), nine (Minicozzi et al. 2017, recoded according to the same grouping) and seventeen cancer registries (Tagliabue et al. 2021) for north, center and south Italy, the latter two studies with only one CR in common.

Therefore, we considered reliable to apply the recent and robust Romagna Unit of the Emilia-Romagna Cancer Registry data on BC subtypes to Italy.

Finally, as a general warning we highlight that any estimate, as the presented ones, may be considered for the order of magnitude, being prone, as explained, to a certain amount of approximation.

In conclusion, the present estimates with clinically relevant information on BC subtypes and therefore therapy, apply for supporting oncologists, decision-makers, and all other stakeholders on the leading cancer for women.

## Data Availability

The datasets generated during and/or analyzed during the current study are available from the corresponding author on reasonable request.
